# Progression of benign prostatic hyperplasia is associated with pro-inflammatory mediators and chronic activation of prostate-infiltrating lymphocytes

**DOI:** 10.18632/oncotarget.8051

**Published:** 2016-03-14

**Authors:** Melissa M. Norström, Emelie Rådestad, Berit Sundberg, Jonas Mattsson, Lars Henningsohn, Victor Levitsky, Michael Uhlin

**Affiliations:** ^1^ Pharmaceutical Sciences (PS), Roche Pharmaceutical Research and Early Development (pRED), Roche Innovation Center, Basel, Switzerland; ^2^ Department of Oncology and Pathology, Karolinska Institutet, Stockholm, Sweden; ^3^ Centre for Allogeneic Stem Cell Transplantation (CAST), Karolinska University Hospital, Huddinge, Sweden; ^4^ Department of Clinical Science, Intervention and Technology (CLINTEC), Karolinska Institute, Stockholm, Sweden; ^5^ Roche Pharmaceutical Research and Early Development (pRED), Roche Innovation Center, Zurich, Switzerland; ^6^ Department of Clinical Immunology and Transfusion Medicine, Karolinska University Hospital, Stockholm, Sweden; ^7^ Department of Applied Physics, Royal Institute of Technology, Stockholm, Sweden; ^8^ Current address: Oncology Research, Molecular Partners AG, Zurich, Switzerland

**Keywords:** benign prostatic hyperplasia, prostate-infiltrating lymphocytes, immunophenotyping, cytokine/chemokine profiling, inflammation

## Abstract

Benign prostatic hyperplasia (BPH) is a common chronic non-malignant condition whose prevalence substantially increases with age. Immune cell infiltration and pro-inflammatory mediators have been implicated in the pathogenesis. Here, we characterized 21 extracellular markers on prostate-infiltrating lymphocytes (PILs) and analyzed expression of 26 soluble proteins in prostate tissue obtained from BPH patients (*n* = 31). These data were correlated with clinical parameters and compared with peripheral blood mononuclear cells (PBMCs) (*n* = 10). Increased frequencies of T cells expressing co-inhibitory receptors LAG-3, PD-1, TIM-3 or CTLA-4, and co-stimulatory receptors CD28, OX40 or 4-1BB were observed in BPH tissue compared to PBMCs. These findings are consistent with chronic activation and possible functional exhaustion of PILs that may be further augmented by several identified pro-inflammatory factors, such as IL-8 and MCP-1, promoting inflammation and chemotaxis of immune cells to the prostate. Prostate size and plasma prostate-specific antigen levels positively correlated with IL-8 and MCP-1 concentrations, and frequencies of T cells expressing CTLA-4 and TIM-3. It remains to be established whether the link between inflammation and BPH progression supported by our findings reflects a progressive failure of the immune system leading to decreased immune surveillance and development of prostate cancer.

## INTRODUCTION

Benign prostatic hyperplasia (BPH) refers to a non-malignant stromal and epithelial cell propagation resulting in enlargement of the prostate. BPH is a chronic progressive condition affecting approximately 10% of men at age of 30, gradually reaching prevalence of 80-90% for 70-80 year olds [1]. The excessive growth can compress the urethra and/or grow into the bladder increasing the risk for a number of symptoms during the emptying and storage phases of micturition [2, 3]. Treatments aimed to reduce symptoms include surgical procedures and/or pharmacological therapy affecting the neural and/or hormonal control of the lower urinary tract [3].

Knowledge regarding the pathogenesis of BPH still remains fragmentary but its progression may result from multiple factors including changes in epithelial-stromal interactions, local endocrine and autonomous nerve system deregulations [2]. Increased infiltration of immune cells and pro-inflammatory factors have previously been found in BPH [1, 2, 4–7]. However, the role of inflammation and the immunological involvement in BPH pathogenesis remains poorly understood.

We performed a detailed comparative phenotypic characterization of prostate-infiltrating lymphocytes (PILs) freshly isolated from BPH tissue and their counterparts isolated from peripheral blood (PB). In addition, soluble factors in the prostate tissue were analyzed to identify pro-inflammatory components which may affect the microenvironment of BPH lesions. The overall goal of the study was to gain knowledge regarding the immunological involvement in BPH progression by correlating clinical parameters of the disease and *in situ* immune activation.

## RESULTS

### Comparing frequencies of immune cell subsets between BPH tissue and PB

We investigated the phenotype of lymphocytes freshly isolated from prostate tissue (*n* = 31) (Table 1) and PB (*n* = 10) of BPH patients. Cell viability of PILs was maintained during processing with a median of 83.9% living cells (Table 2).

**Table 1:** Patient characteristics and sample information of obtained benign prostatic hyperplasia (BPH) tissue (*n* = 31)

Patient nr procedure	Age (yr)	Resected weight (g)	Collected weight (g)	Prostate size (g)		P-PSA (μg/L)		5-ARI	Alpha- blocker	Urine bacteria culture	Pathology report
1 TURP	83	4.3	2.3	N/A	-	127	❸	Yes	No	*Aerococcus urinae*	BPH
2 TURP	66	34	5	85	②	11	❸	No	No	*Negative*	BPH
3 TURP	81	5	2.3	45	①	5	❷	No	No	*Negative*	BPH
4 TURP	92	10	2	32	①	0.6	❶	No	No	*Negative*	BPH
5 TURP	64	20	5.2	62	②	3.8	❶	No	No	*Negative*	BPH
6 TURP	68	20	10	37	①	3.3	❶	No	No	*Klebsiella oxytoca*	BPH
7 TURP	75	15	5	42	①	3.4	❶	No	No	*Enterococcus faecalis*	BPH
8 TURP	56	25	5.1	70	②	20	❸	No	N/A	*N/A*	BPH
9 TURP	68	30	10	65	②	9	❷	Yes	No	*Negative*	BPH
10 TURP	93	22	10	57	①	N/A	-	No	No	*Enterococcus*	BPH, PC
11 TURP	77	27	6	33	①	1	❶	Yes	Yes	*Negative*	BPH
12 TURP	62	32	10	54	①	N/A	-	No	No	*Staph. epidermidis*	BPH
13 TURP	66	35	10	61	②	7.1	❷	N/A	Yes	*Enterobacter*	BPH, PC
14 TURP	66	35	10	62	②	11	❸	N/A	N/A	*Enterobacter cloacae*	BPH
15 TURP	83	7	3	37	①	N/A	-	N/A	No	*N/A*	BPH
16 TURP	77	15	7	40	①	N/A	-	Yes	No	*Negative*	BPH
17 TURP	75	30	10	35	①	0.8	❶	Yes	No	*Staph. aureus*	BPH
18 TURP	75	48	10	65	②	11	❸	No	Yes	*Negative*	BPH, PC
19 TURP	74	26	10	37	①	5	❷	No	Yes	*Negative*	BPH, PC
20 TURP	74	23	5.2	N/A	-	5.6	❷	N/A	No	*Negative*	BPH
21 TURP	72	20	8.2	30	①	N/A	-	Yes	No	*E. coli*	BPH
22 TURP	66	8	3	N/A	-	N/A	-	No	No	*Negative*	BPH
23 TURP	62	25	10	48	①	4.4	❶	Yes	No	*Negative*	BPH
24 TURP	63	32	10	66	②	4	❶	N/A	No	*Negative*	BPH
25 TURP	77	36	10	83	②	6	❷	No	No	*Acinetobacter*	BPH
26 AE	71	87	15	115	②	8.7	❷	No	No	*Negative*	BPH
27 AE	66	84	27	120	②	150	❸	No	No	*Negative*	BPH
28 AE	79	92	21	180	②	N/A	-	No	No	*E. coli ESBL*	BPH
29 AE	69	144	13	N/A	-	N/A	-	Yes	No	*E. coli*	BPH
30 AE	78	98	7.5	107	②	4	❶	Yes	Yes	*Proteus mirabilis*	BPH
31 AE	63	N/A	3.5	130	②	3.8	❶	Yes	No	*E. coli*	BPH
**Median** (min-max)	**72** (56-93)	**27** (5-144)	**10** (2-27)	**61** (30-180)		**5** (0.6-150)					

**Abbreviations:** TURP, transurethral resection of the prostate; AE, adenoma enucleation; P-PSA, plasma prostate-specific antigen; 5-ARI, 5α-reductase inhibitors; N/A, not available; PC, prostate cancer Gleason score 3+3; Staph, Staphylococcus; E. Coli, Escherichia coli; ESBL, extended spectrum beta-lactamase. Clinical groupings: prostate size (① ≤61g and ②>61g); p-PSA (❶<4.5 μg/L; ❷4.5-10 μg/L; ❸>10 μg/L).

**Table 2:** Median frequencies (%) of general immune cell subsets in peripheral blood (PB) (*n* = 10) and benign prostatic hyperplasia (BPH) tissue (*n* = 31)

Cell subset	PB	BPH tissue	Change (in tissue)	>P-value
Total viability	**78.3** (66.9-84.9)	**83.9** (43.1-96.9)	-	ns
Total CD3^−^	**43.5** (23.0-71.0)	**82.1** (52.3-98.2)	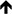	<0.0001
B cells	**5.0** (2.8-16.8)	**2.3** (0.2-37.0)	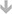	0.010
NK cells	**77.9** (39.7-86.8)	**6.3** (0.3-59.7)	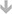	<0.0001
Total CD3^+^	**56.3** (28.9-77.0)	**17.0** (1.4-46.6)	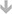	<0.0001
CD4/CD8 ratio	**1.7** (0.6-4.2)	**0.6** (0.4-6.1)	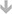	0.005
CD4^+^	**52.0** (32.9-71.9)	**34.7** (23.9-77.8)	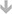	0.009
CD8^+^	**30.9** (17.3-62)	**57.4** (12.7-66.2)	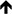	0.012
T_N_	**15.2** (3.5-39.7)	**4.7** (0-12.6)	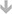	<0.0001
T_CM_	**15.5** (7.7-46.3)	**10.3** (0-38.4)	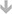	0.028
T_EM_	**31.6** (20.4-47.0)	**46.0** (15.0-83.3)	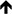	0.010
T_TD_	**29.5** (7.6-53.4)	**37.9** (5.1-63.1)	-	ns
Treg	**7.2** (3.0-11.0)	**16.6** (10.8-33.0)	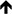	<0.0001

Values are presented as median frequencies (min/max). Arrows indicate significant frequency change of presented T cell subset populations in BPH tissue compared to PB; 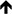, increased frequency in BPH tissue; 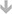, decreased frequency in BPH tissue; -, no difference. **Abbreviations:** ns, no significance; B cells (gated from CD3^−^/CD19^+^); NK, natural killer cells (gated from CD3^−^/CD56^+^); T_N_, naïve T cells (CCR7^+^CD45RO^−^); T_CM_, central memory T cells (CCR7^+^CD45RO^+^); T_EM_, effector memory T cells (CCR7^−^CD45RO^+^); T_TD_, terminally differentiated T cells (CCR7^−^CD45RO^−^); Treg, potential regulatory T cells (gated from CD4^+^ /CD25^high^CD127^−/low^)

**Figure 1:**
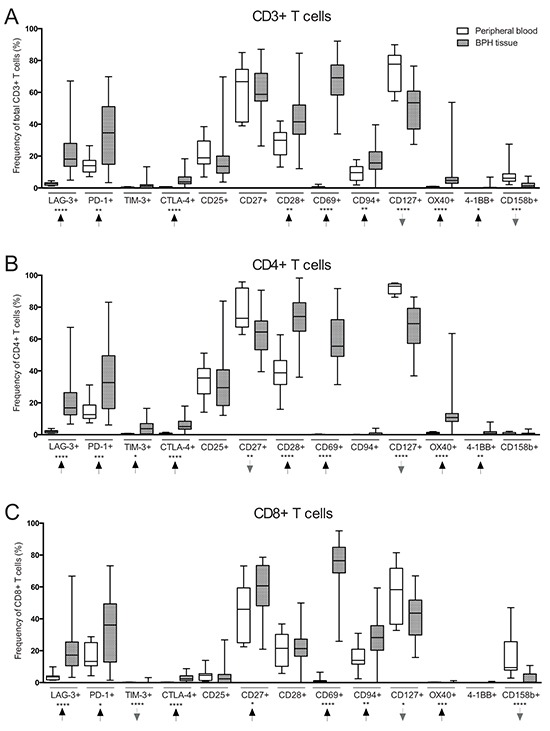
Comparison of T cell subset frequencies in peripheral blood (PB) (*n* = 10) and benign prostatic hyperplasia (BPH) tissue (*n* = 31) obtained from BPH patients **A.** Frequency (%) of CD3^+^ (total) T cells expressing the presented markers on the X-axis. **B.** Frequency of CD4^+^ T cells expressing the presented markers. **C.** Frequency of CD8^+^ T cells expressing the presented markers. Arrows indicate frequency change in BPH tissue compared to PB. Graphs show box plots with median, interquartile range and min/max values. Significances are presented as *p≤0.05, **p≤0.01, ***p≤0.001 and ****p≤0.0001.

Comparing peripheral blood mononuclear cells (PBMCs) with PILs from BPH patients showed major differences in composition of analyzed immune cell subsets (Figure 1, Table 2 and Table 3). There was an increased frequency of CD3^−^ cells but decreased frequencies of B cells and natural killer (NK) cells in BPH tissue compared to PBMCs (Table 2). Subdividing CD3^+^ T cells into CD4^+^ and CD8^+^ T cells revealed a strong decrease of CD4:CD8 ratio in BPH tissue compared to PBMCs (0.6 *vs*. 1.7) (Table 2). In approximately 75% of the obtained BPH tissue samples (*n* = 23), there were more CD8^+^ T cells present than CD4^+^ T cells.

There were significantly reduced proportions of naïve (CCR7^+^CD45RO^−^) and central memory T cells (CCR7^+^CD45RO^+^) in BPH tissue as compared to PBMCs. The proportion of effector memory T cells (CCR7^−^CD45RO^+^) was increased while there was no difference in terminally differentiated T cells (CCR7^−^CD45RO^−^) (Table 2). Furthermore, there was a substantially increased presence of potential regulatory T cells (CD4^+^/CD25^high^CD127^−/low^) in BPH tissue as compared to PBMCs (median 16.6% *vs*. 7.2%, p<0.0001) (Table 2).

T cells expressing co-inhibitory receptors lymphocyte-activation gene-3 (LAG-3), programmed cell death-1 (PD-1), T cell immunoglobulin mucin-3 (TIM-3) or cytotoxic T lymphocyte-associated protein-4 (CTLA-4) were increased in frequency in BPH tissue in all T cell compartments (total CD3^+^ T cells, CD4^+^ and CD8^+^ T cells) as compared to PBMCs. CD8^+^ T cells expressing TIM-3 were almost completely absent in both PB and BPH tissue (Figure 1 and Table 3).

**Table 3:** Median frequencies (%) of T cell subsets expressing different markers in peripheral blood (PB) (*n* = 10) and benign prostatic hyperplasia (BPH) tissue (*n* = 31)

Subset	-----CD3^+^ T cells (%)-----				-----CD4^+^ T cells (%)-----				-----CD8^+^ T cells (%)-----			
Marker	PB	BPH tissue	Change (in tissue)	P-value	PB	BPH tissue	Change (in tissue)	P-value	PB	BPH tissue	Change (in tissue)	P-value
LAG-3^+^	2.7	18.1	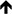	<0.0001	2.1	16.9	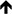	<0.0001	3.6	17.2	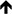	<0.0001
PD-1^+^	14.0	34.6	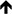	0.008	12.6	32.7	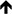	0.001	13.3	36.1	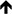	0.025
TIM-3^+^	0.3	1.2	-	ns	0.4	3.8	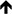	0.012	0.2	0	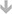	<0.0001
CTLA-4^+^	0.6	3.8	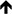	<0.0001	0.8	5.1	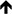	<0.0001	0.2	2.4	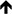	<0.0001
CD25^+^	18.9	13.6	-	ns	35.6	29.5	-	ns	4.6	2.4	-	ns
CD27^+^	66.7	58.7	-	ns	73.0	64.4	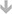	0.004	46.0	60.7	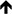	0.023
CD28^+^	30.0	41.5	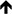	0.002	38.8	74.0	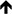	<0.0001	21.5	21.3	-	ns
CD69^+^	0.6	69.2	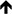	<0.0001	0.2	55.5	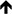	<0.0001	0.8	76.5	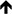	<0.0001
CD94^+^	9.7	15.7	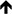	0.006	0.1	0	-	ns	13.9	28.3	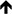	0.005
CD127^+^	77.7	53.5	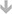	<0.0001	93.2	69.6	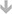	<0.0001	58.3	43.6	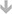	0.034
OX40^+^	0.6	4.7	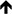	<0.0001	0.8	10.7	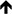	<0.0001	0.2	0	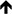	0.0001
4-1BB^+^	0.0	0.0	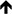	0.026	0.0	0.8	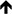	0.006	0	0	-	ns
CD158b^+^	6.2	1.3	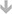	0.0002	0.5	0.4	-	ns	9.5	0.3	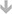	<0.0001

Values are presented as median. Arrows indicate significant frequency change of presented T cell subset populations in BPH tissue compared to PB. Frequency change in BPH tissue compared to PB; 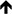, increased frequency in BPH tissue; 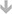, decreased frequency in BPH tissue; -, no difference. **Abbreviations**: PB, peripheral blood; BPH, benign prostatic hyperplasia; ns, no significance

T cells of all compartments expressing co-stimulatory receptors CD28, OX40 or 4-1BB were increased in frequency in BPH tissue as compared to PBMCs (Figure 1 and Table 3). CD4^+^ T cells expressing these markers were more abundant than CD8^+^ T cells. There was no difference in frequency of total T cells expressing co-stimulatory receptor CD27, but CD4^+^/CD27^+^ were found decreased in frequency in BPH tissue as compared to PBMCs (64.4% *vs*. 73.0%, p=0.004) while CD8^+^/CD27^+^ were increased in BPH tissue (60.7% *vs*. 46.0%, p=0.023) (Figure 1 and Table 3). There was a pronounced increase in frequency of total T cells expressing CD69 in BPH tissue (0.6% *vs*. 69.2%, p=<0.0001), and CD69 was expressed more frequently by CD8^+^ T cells than CD4^+^ T cells (76.5% *vs*. 55.5% in BPH tissue) (Figure 1 and Table 3). Frequency of CD8^+^ T cells expressing CD94 were also increased in BPH tissue compared to PBMCs (Figure 1 and Table 3).

In contrast, the frequency of T cells expressing CD127 was decreased in BPH tissue as compared to PBMCs. CD127 expression was primarily found on CD4^+^ T cells in both BPH tissue and PBMCs (69.6% CD4^+^/CD127^+^ *vs*. 43.6% CD8^+^/CD127^+^ in BPH tissue) (Figure 1 and Table 3). Decreased presence of T cells expressing CD158b was also observed, significant for total T cells and CD8^+^ T cells (Figure 1 and Table 3).

Frequency of T cells expressing CD25 did not differ between BPH tissue and PBMCs in any T cell compartment. Expression was observed predominantly by CD4^+^ T cells compared to CD8^+^ T cells in both BPH tissue and PBMCs (29.5% CD4^+^/CD25^+^ *vs*. 2.4% CD8^+^/CD25^+^ in BPH tissue) (Figure 1 and Table 3).

### Prostate-infiltrating T cell subsets and clinical groupings

Patients were subdivided based on clinical parameters to elucidate potential reasons for the observed significant heterogeneity in T cell subset composition in BPH tissue between the patients. A positive correlation was found between prostate size and plasma prostate-specific antigen (p-PSA) level (p=0.003, r=0.610).

#### Prostate size correlates with increased presence of T cells expressing CTLA-4

**Figure 2:**
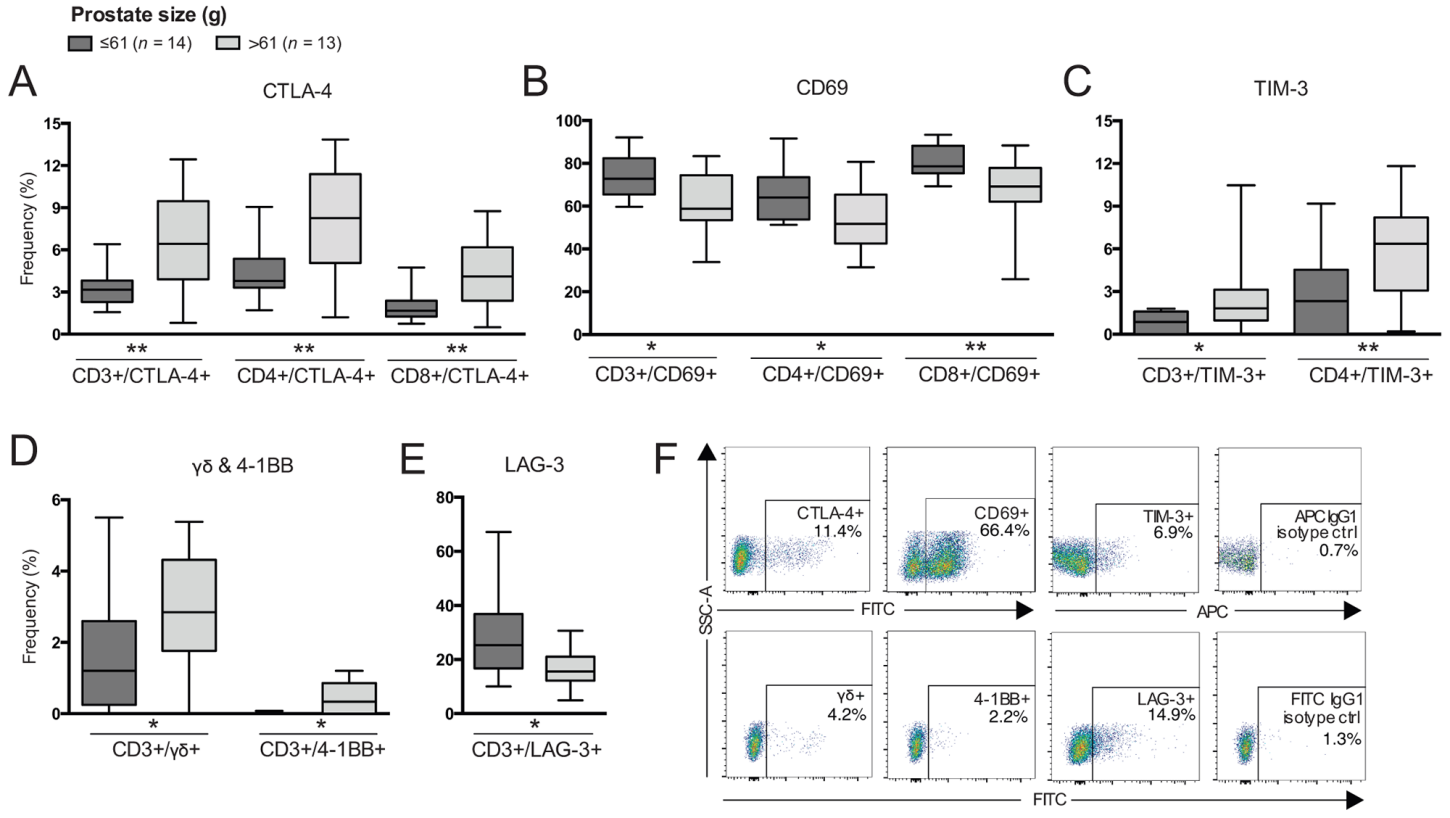
Differences in frequencies of T cell subsets comparing patients based on prostate size Frequency (%) of CD3^+^ (total T cells), CD4^+^ and/or CD8^+^ T cells expressing: **A.** CTLA-4; **B.** CD69; **C.** TIM-3; **D.** γδ and 4-1BB; and **E.** LAG-3. **F.** Representative plots of presented cell populations gated from CD3^+^ T cells with corresponding isotype control. Significances are presented as *p≤0.05 and **p≤0.01.

Patients with larger prostate size (>61 g) had an increased frequency of all T cell compartments expressing CTLA-4 compared to those with smaller prostate size (≤61 g) (Figure 2A). There was a positive correlation between prostate size and frequency of total T cells expressing CTLA-4 (p=0.004, r=0.543).

In contrast, frequencies of all compartments of T cells expressing CD69 were decreased in patients with larger prostate size (Figure 2B) and a negative correlation was determined (p=0.008, r=−0.497). Increased prostate size was also associated with increased frequency of TIM-3^+^ T cells, γδ T cells and 4-1BB^+^ T cells (Figure 2C and 2D) and these populations also positively correlated with prostate size by Spearman's correlation (data not shown). Furthermore, frequency of total T cells expressing LAG-3 was decreased in patients with larger prostate size (Figure 2E). A negative correlation was also determined between total T cells expressing LAG-3 and prostate size (p=0.040, r=−0.398). Additionally, frequency of CD4^+^ T cells expressing LAG-3 showed a trend in being reduced, however, not significant (p=0.06, data not shown). Representative plots are presented in Figure 2F.

#### P-PSA correlates with frequency of T cells expressing 4-1BB and TIM-3

**Figure 3:**
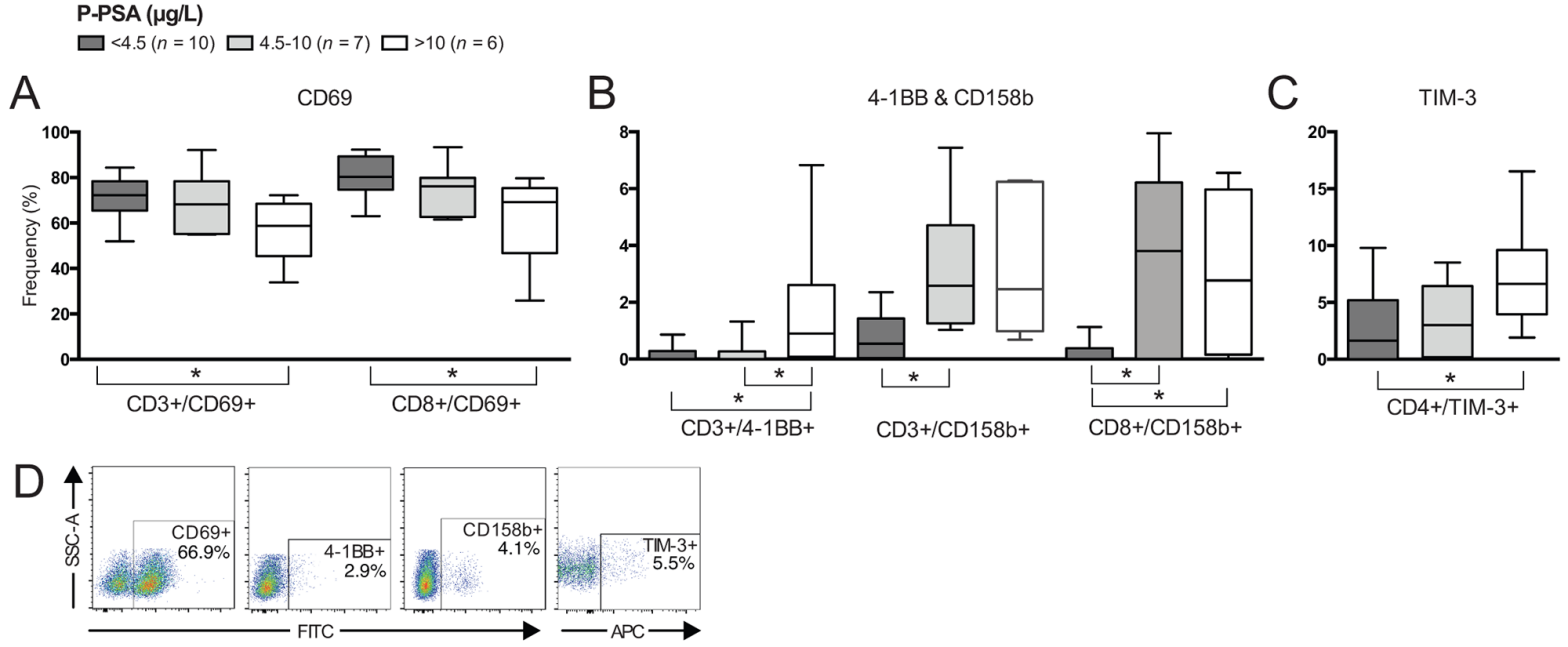
Differences in frequencies of T cell subsets comparing patients based on plasma prostate-specific antigen (p-PSA) level Frequency (%) of CD3^+^ (total T cells), CD4^+^ and/or CD8^+^ T cells expressing: **A.** CD69; **B.** 4-1BB and CD158b; and **C.** TIM-3. **D.** Representative plots of presented cell populations gated from CD3^+^ T cells. Significances are presented as *p≤0.05.

Frequency of total and CD8^+^ T cells expressing CD69 was decreased in patients with high p-PSA (>10 μg/L) compared to patients with low p-PSA (<4.5 μg/L) (Figure 3A), also showing a negative correlation between frequency of CD69-expressing total T cells and p-PSA (p=0.011, r=−0.531). A similar pattern was observed with CD4^+^ T cells expressing CD69 but not significant (p=0.054). Frequency of total T cells expressing 4-1BB was limited in patients with low and intermediate (>4.5-10 μg/L) p-PSA but statistically increased in patients with high p-PSA (Figure 3B), and a positive correlation with p-PSA was determined (p=0.026, r=0.475).

CD158b-expressing total T cells were increased in frequency in patients with intermediate p-PSA and high p-PSA compared to patients with low p-PSA (Figure 3B) and there was a positive correlation between p-PSA and CD158b-expressing T cells (p=0.050, r=0.457). CD4^+^ T cells expressing TIM-3 were increased in frequency in patients with high p-PSA compared to those with low p-PSA (Figure 3C). A positive correlation between p-PSA and CD4^+^/TIM3^+^ frequency was also determined (p=0.015, r=0.511). Representative plots are presented in Figure 3D.

#### Presence of malignancy correlates with increased frequency of LAG-3^+^ T cells

**Figure 4:**
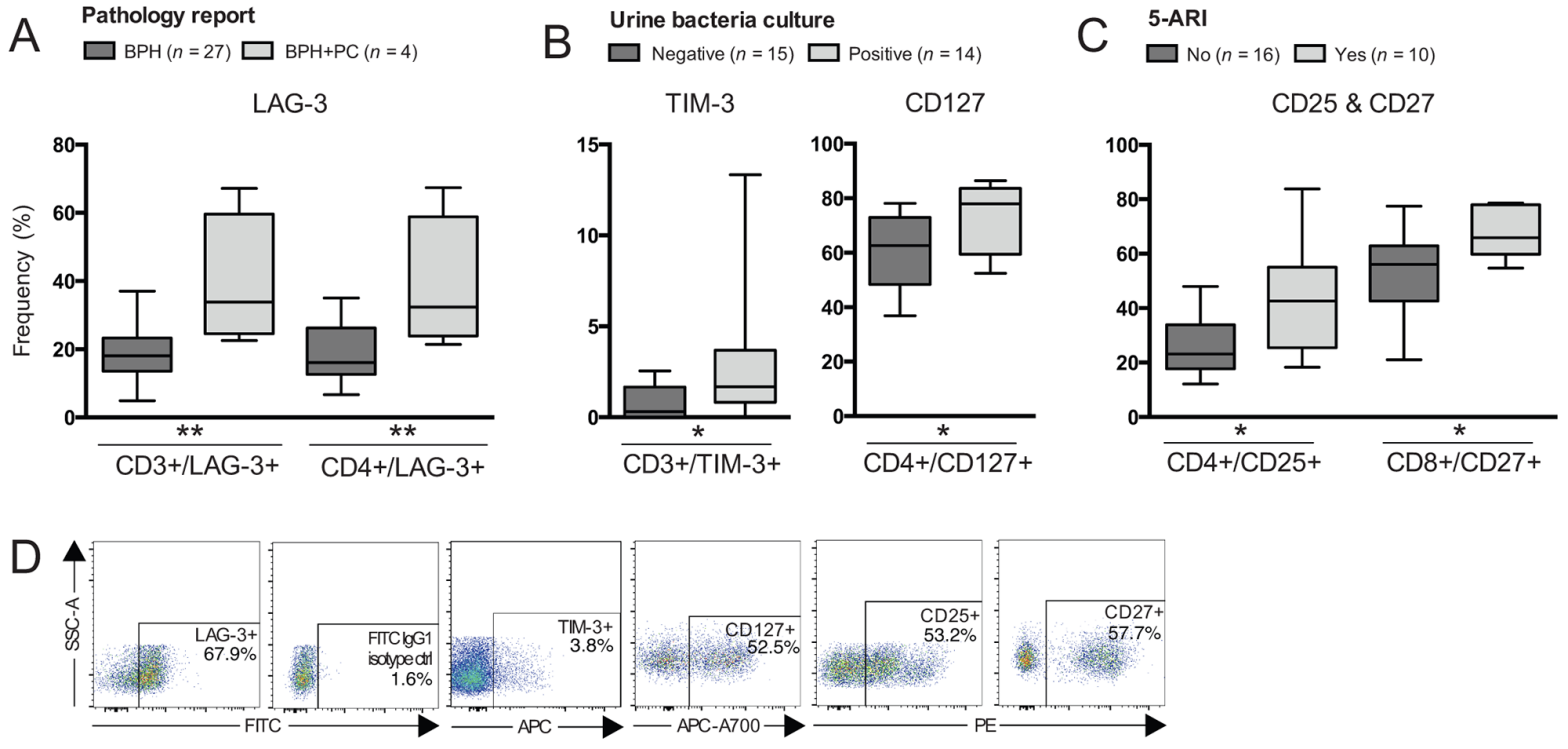
Differences in frequencies of T cell subsets comparing patients based on other clinical groupings Patients were grouped according to: **A.** Pathology report; **B.** Urine bacteria culture; or **C.** Treatment with 5α-reductase inhibitors (5-ARI). Frequency (%) of CD3^+^ (total T cells), CD4^+^ and/or CD8^+^ T cells expressing: A. LAG-3; B. TIM-3 and CD127; C. CD25 and CD27. **D.** Representative plots of presented cell populations gated from CD3^+^ T cells. Significances are presented as *p≤0.05 and **p≤0.01.

Four patients histologically diagnosed with BPH and prostate cancer (PC) showed an increased frequency of total T cells expressing LAG-3 compared to patients without histological signs of malignancy (*n* = 27) (p=0.006). The same pattern was observed with CD4^+^ T cells expressing LAG-3 (Figure 4A) (p=0.009).

Patients with a urinary tract infection had an increased frequency of total T cells expressing TIM-3 and CD4^+^ T cells expressing CD127 compared to patients with a negative bacteria culture result (Figure 4B) (p=0.035 and p=0.025 respectively).

Patients treated with 5α-reductase inhibitors (5-ARI) had an increased frequency of CD4^+^ T cells expressing CD25 compared to patients without treatment (Figure 4C) (p=0.023). The same finding was observed with CD8^+^ T cells expressing CD27 (Figure 4C) (p=0.014). Representative plots are presented in Figure 4D.

### Cytokine and chemokine profiling of BPH tissues

**Figure 5:**
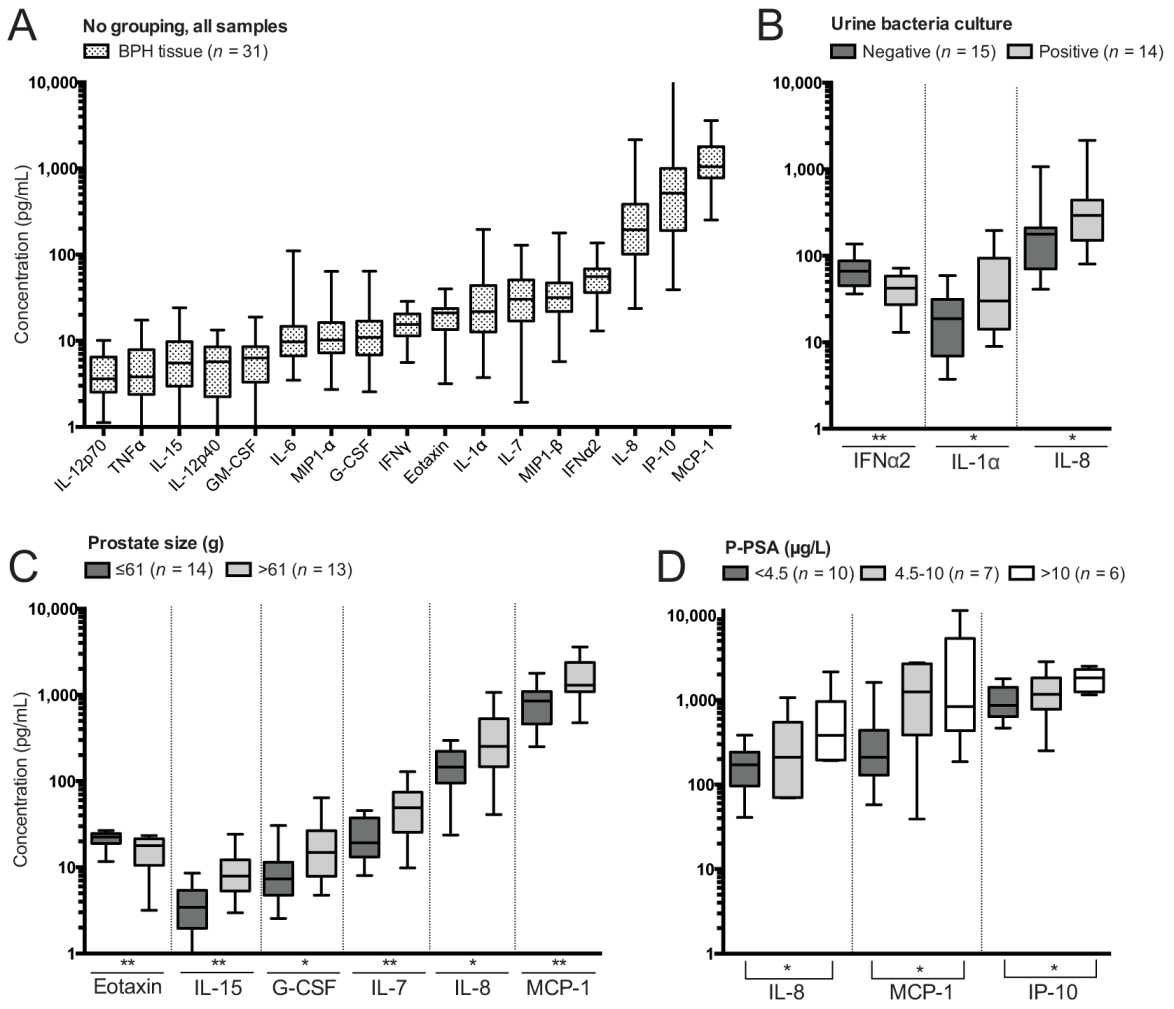
Soluble protein concentrations determined by Luminex in supernatants of benign prostatic hyperplasia (BPH) tissue processing and differences comparing clinical groupings **A.** Concentrations of soluble proteins in all BPH tissue samples. **B.** Significant differences in concentrations based on subgrouping patients on results of urine bacteria culture. **C.** Significant differences in concentrations based on subgrouping patients on prostate size. **D.** Significant differences in concentrations based on subgrouping patients on levels of plasma prostate-specific antigen (p-PSA). Presented significances are between the low and high group as indicated by the line. Note that concentration plotted on the y-axis has a logarithmic scale and that proteins have been plotted hierarchically based on their concentration. Significances are presented as *p≤0.05 and **p≤0.01.

Analysis of 26 soluble proteins in supernatants collected during BPH tissue processing was performed using multiplex immunoassay. Nine cytokines (IL1-β, IL-2, IL-3, IL-4, IL-5, IL-10, IL-13, IL-17A and TNF-β) were excluded as being undetectable in the analyzed samples, and the remaining are presented in Figure 5A. No differences were identified comparing patients based on pathology report, treatment with 5-ARI or α-adrenergic receptor blockers (α-blockers). Patients with a urinary tract infection had decreased levels of IFN-α2 in the prostate while having increased levels of IL-1α and IL-8 compared to patients with a negative urine culture (Figure 5B).

Granulocyte-colony stimulating factor (G-CSF), monocyte chemotactic protein-1 (MCP-1), IL-7, IL-8 and IL-15 were increased in BPH tissue of patients with larger prostate size, while the concentration of eotaxin was reduced compared to patients with lower prostate size (Figure 5C). Prostate size was positively correlated to all of these factors except eotaxin, which was negatively correlated (Supplementary Figure S1A).

IL-8, MCP-1 and interferon gamma-induced protein-10 (IP-10) were elevated in BPH tissue of patients with high p-PSA compared to patients with low p-PSA (Figure 5D). There were also positive correlations between p-PSA and all of these soluble proteins (Supplementary Figure S1B). In addition, there was a trend towards reduced eotaxin in patients with high p-PSA compared to those with low p-PSA (p=0.0502). However, a negative correlation between p-PSA and eotaxin was significant (p=0.031, r=0.472, Supplementary Figure S1B).

## DISCUSSION

The role of the immune system in the pathogenesis in BPH is uncertain, but prostatic inflammation is commonly found in BPH patients [7, 8]. Earlier studies have suggested chronic activation of lymphocytes in BPH tissue based on their expression of HLA-DR and CD25 [9, 10], but limitations of the methods available at the time call for an updated in-depth analysis of lymphocytes present in BPH. In this study, we performed a detailed characterization comparing lymphocytes freshly isolated from BPH tissue with their PB counterparts and measured numerous soluble factors.

We found indications of chronic activation supported by presence of T cells expressing activation-induced co-inhibitory receptors, co-stimulatory receptors and high frequency of potential regulatory T cells in BPH tissue. Previous studies have shown that BPH-derived epithelial and stromal cells produce pro-inflammatory cytokines and chemokines [6, 11, 12]. Here, we confirm presence of abundant soluble pro-inflammatory factors in BPH-affected tissue. We found a correlation between prostate size and p-PSA in accordance with previous studies [13, 14]. Furthermore, prostate size and p-PSA were associated with several lymphocyte subsets and soluble factors highlighting possible involvement in the progression of BPH.

In the current study, lymphocytes isolated from BPH tissue differed greatly in subset composition compared to lymphocytes isolated from PB. This is to some extent expected as the role of the immune system differs in these different bodily environments. Regarding CD3^+^ T cell subgrouping into CD4^+^ and CD8^+^ T cells, we found a clear predominance of CD8^+^ T cells in BPH tissue consistent with previous findings in a large cohort of BPH specimens [15]. In the same study, differences in CD4/CD8 composition in different locations of the prostate were suggested [15], possibly explaining the contradicting results of two other studies which have reported predominance of CD4^+^ T cells [9, 10].

Our results revealed increased frequencies of T cells expressing co-inhibitory receptors (LAG-3, PD-1, TIM-3, and CTLA-4) in BPH tissue compared to PBMCs; T cells expressing PD-1 being the most abundant subset. These co-inhibitory receptors are up-regulated in response to activation, and through different pathways they dampen and control an active immune response [16–18]. Increased frequency of T cells expressing these receptors could suggest a recent, but more likely a chronic, activation of T cells in the prostate environment. Upon binding with its corresponding ligands, the consequence of the expression of these co-inhibitory receptors could be exhaustion or anergy associated with loss of function in terms of cytokine production, capacity to proliferate, and perform effector functions [19, 20].

Interestingly, frequencies of several lymphocyte subsets expressing co-inhibitory receptors correlated with clinical parameters. Increasing prostate size and/or p-PSA can be seen as signs of long BPH development [3] and, speculatively, a long ongoing chronic activation of lymphocytes in the prostate resulting in up-regulation of these co-inhibitory receptors. In the case of TIM-3, increased frequency of T cells expressing this receptor also correlated with presence of urinary infection making the interpretation of the involvement in chronic or recent activation in BPH tissue difficult. Increased prostate size also correlated with a decreased frequency of total T cells expressing LAG-3, further complicating the link between clinical parameters and co-inhibitory receptor expression.

Despite the low number of patients diagnosed with malignancy (*n* = 4), T cells expressing LAG-3 were significantly elevated in frequency in these patients. Speculatively, this could be a mechanism of immune evasion by tumor cells and warrants further studies elucidating the prevalence of LAG-3 expression in prostate tissue of PC patients. In a study by Sfanos and colleagues, high frequency of PD-1^+^CD8^+^ T cells was reported [21]. Antibody-based checkpoint-blockade therapy is gaining increasing interest and a phase I clinical trial focusing on blocking LAG-3 with or without simultaneous blocking of PD-1 in solid tumors is currently ongoing (www.ClinicalTrials.gov, NCT01968109). Additional profiling of co-inhibitory receptors in PC is warranted for predictive purposes in future treatments utilizing these immunotherapeutic approaches.

Androgen-depleting treatment with 5-ARI was associated with increased frequency of CD4^+^ T cells expressing CD25 (α chain of the IL-2 receptor) and CD8^+^ T cells expressing co-stimulatory receptor CD27.This medication effectively limits the amount of available dihydrotestosterone (DHT), a more potent form of testosterone, in blood and prostate. Based on functional assays, it has been suggested that DHT acts as an anti-inflammatory factor for CD4^+^ T cells, inhibiting their IL-2 production [22]. With our results, we illustrate that it is possible that 5-ARI treatment alters the phenotype of immune cells present in the prostate, increasing their capacity to become activated.

The concentrations of several pro-inflammatory factors were found to be correlated to clinical parameters, in particular prostate size and p-PSA. Elevated serum-PSA levels have previously been shown to positively correlate with presence and extent of inflammation [23, 24]. Here, prostate size and p-PSA were found to correlate with IL-8 and MCP-1, which have previously been suggested to be involved in BPH progression [6, 11, 12, 25, 26]. Based on *in vitro* assays, IL-8 and MCP-1 can be produced by epithelial and/or stromal cells of the prostate [6, 11, 12]. It is possible that they stimulate epithelial/stromal cell growth, thereby causing increased prostate tissue growth [12, 25]. In support, IL-8 and MCP-1 have also been correlated with prostate weight [6, 25]. The chemotactic role of MCP-1 could be of importance in the context of BPH, further enhancing an ongoing inflammatory response. Our results confirm increased presence of IL-8 and MCP-1 with increased prostate size and levels of p-PSA, further enhancing support for their involvement in BPH pathogenesis.

Vignozzi *et al.* [22] have shown that co-culturing of BPH stromal cells and CD4^+^ T cells resulted in secretion of multiple soluble proteins; IL-6, IL-8, IP-10, MCP-1, G-CSF and eotaxin being found most abundant. In addition, Penna *et al.* [11] have suggested a positive feedback loop for hyperplasia progression and amplification of inflammation caused by secretion of IL-6, IL-8, and IP-10 from BPH stromal cells. These studies identified pro-inflammatory soluble factors based on *in vitro* culturing studies. Here, we confirm presence of these proteins *in vivo* and correlated them with prostate size and/or p-PSA.

In conclusion, BPH tissue was found to be a pro-inflammatory, chemotactically attractive site in which chronic activation could induce exhaustion of prostate-infiltrating T cells. There is currently no direct evidence that BPH is a precursor of malignant transformation [1, 27]. However, progressively exhausted immune cells in BPH sites could suppress active surveillance of malignant transformation. It remains to be established whether the link between the inflammatory milieu and BPH progression supported by our findings reflects a progressive failure of the infiltrating immune cells leading to decreased immune surveillance. As chronic inflammation is increasingly thought to be involved in development of PC [8, 28, 29], it is crucial to obtain deeper knowledge about the BPH-associated inflammation process and its implications for the functionality of PILs. Our data provide important information regarding the phenotype of lymphocyte populations and the composition of soluble pro-inflammatory factors present in BPH tissue.

Ideally, the results of the current study should have been strengthened by comparative analysis of healthy prostate tissue. This was, however, not possible due to ethical constrains and logistic limitations. Nevertheless, this matter needs to be addressed in follow-up studies. Detailed characterization of infiltrating immune cells and the microenvironment in healthy, BPH and malignant prostate tissue could help to develop new approaches to prophylactic and therapeutic treatment of prostate cancer.

## MATERIALS AND METHODS

### Ethics statement

This study has been conducted in accordance with the Declaration of Helsinki, according to national/international guidelines, and was approved by the Regional Ethical Review Board in Stockholm, Sweden (2010/158-31/2, 2013/212232). Patients were informed about the study and gave their consent if willing to participate.

### Patients and sample collection

BPH tissue was obtained from 31 patients at the end of transurethral resection of the prostate (TURP) (*n* = 25) or adeno enucleation (AE) (*n* = 6) surgical procedure at Karolinska University Hospital Huddinge, Sweden (Table 1). A median of 27 g of prostate tissue was resected, and a median of 10 g of tissue was collected for the current study during the procedure (Table 1). Collected tissue was immediately placed in PBS (0.01M) and processing began within 15 minutes after collection. The remaining resected prostate tissue was sent to the pathology lab for routine histopathological diagnosis. A total of 20 mL of PB was collected in heparinized vacutainer tubes from ten additional BPH patients (median age 73 years, range 65-84 years, data not shown).

### Patient characteristics and grouping

Clinical parameters were used to subgroup the patients (Table 1). Prostate size was defined as estimated weight of the prostate (in grams) prior to surgery as determined by transrectal ultrasound, and patient groups were based on the median of the complete patient group, 61 g (≤61 g, *n* = 14; and >61 g, *n* = 13). P-PSA level was measured in μg/L and patients were divided into three groups: low <4.5 μg/L (*n* = 10); intermediate >4.5-10 μg/L (*n* = 7); and high >10 μg/L (*n* = 6). Grouping based on androgen-depleting treatment with 5-ARI, created two groups; yes (*n* = 16) and no (*n* = 10). Grouping based on treatment with α-blockers created two groups; yes (*n* = 5) and no (*n* = 24), but did not result in any significances. Presence of urinary tract infection was determined by urine bacteria culture prior to surgery and created two groups; patients with a positive (*n* = 15) or negative (*n* = 14) culture. Based on pathology report, four patients were histologically diagnosed with BPH and PC (Gleason score 3+3 for all). These patients were compared with patients without malignancy (*n* = 27).

### Sample processing and isolation of PILs

Isolation of PILs was performed using a recently described protocol involving immediate tissue processing without any enzymatic treatment or long incubation steps, and performing acquisition of fresh cells [30]. Briefly, a gentleMACS Dissociator (Miltenyi Biotec, Bergisch Gladbach, Germany) was used to process the tissue. Thereafter, the tissue suspension was filtered using 70μm nylon cell strainers (BD Biosciences, Franklin Lakes, NJ, USA) and a single cell suspension was obtained. The suspension was centrifuged at 400*g* for 7 min (Rotina 420, Hettich, Tuttlingen, Germany), and supernatant was decanted, collected and stored at −80°C until time of multiplex analysis. The cell pellet was resuspended in PBS and mononuclear cells were isolated using density gradient centrifugation with Lymphoprep (1.077 g/cm^2^, Fresenius Kabi, Oslo, Norway) as previously described [31].

### Processing of PBMCs

PBMCs from blood samples were separated using Lymphoprep as described above. Cells were frozen in complete RPMI medium with 10% DMSO and stored in liquid nitrogen. At day of analysis, PBMCs were thawed in complete RPMI and washed twice with PBS. The complete 1640 RPMI medium (Thermo Scientific, Waltham, MA, USA) contained 2mM L-glutamine (Invitrogen, Carlsbad, CA, USA), 100 U/mL penicillin G (Thermo Scientific), 100μg/mL streptomycin (Thermo Scientific) and 10% human AB serum (Karolinska University Hospital Huddinge).

### Extracellular antibody staining

Cells were stained with titrated antibodies in an extracellular nine-color flow cytometry panel and incubated for 15 min in the dark at 4°C. All included antibodies were monoclonal and of mouse origin. Fluorescein isothiocyanate (FITC)-conjugated anti-CD28 (CD28.1); anti-CD69 (FN50); anti-CD94 (HP-3D9); anti-CD134/OX40 (ACT35); anti-CD158b (CH-L); phycoerythrin (PE)-conjugated anti-CD25 (M-A251); anti-CD27 (M-T271); anti-CD56 (NCAM16.2); anti-CD279/PD-1 (MIH4); PE-CF594-labeled anti-CD197/CCR7 (150503); PE-Cy7-conjugated anti-CD3 (SK7); allophycocyanin (APC)-conjugated anti-CD19 (HIB19); anti-CD45RO (UCHL1); Alexa700 (A700)-conjugated anti-CD56 (B159); APC-Cy7-conjugated anti-CD8 (SK1); V500-conjugated anti-CD4 (RPA-T4); and isotype controls for FITC, PE and APC IgG1 (X40) were purchased from BD Biosciences. FITC-conjugated anti-TCRγδ pan (IMMU510); APC-A700-conjugated anti-CD127 (R34.34) and krome orange-conjugated anti-CD4 (13B8.2) were purchased from Beckman Coulter (Fullerton, CA, USA). FITC-conjugated anti-CD137/4-1BB (4B4-1), anti-CD152/CTLA-4 (A3.4H2.H12), anti-CD223/LAG-3 (17B4), and APC-conjugated anti-CD366/TIM-3 (F38-2E2) were purchased from LifeSpan Biosciences (Seattle, WA, USA). Isotype control for FITC IgG2a (MOPC-173) was purchased from Biolegend (San Diego, CA, USA).

After cell surface staining, cells were centrifuged for 4 min at 700*g* and washed with PBS once. Viability dye 7-aminoactinomycin D (7AAD, BD Biosciences) was added according to manufacturer's instructions and incubated at room temperature in the dark for 10 min. PBS was added to dilute the cell suspension 1:4.

### Data acquisition by multicolor flow cytometry

Cells were acquired on a BD Canto I SORP and BD FACSAria using BD FACSDiva Software v.7.0 and v.6.1.3 respectively (BD Biosciences). Data was analyzed and displayed using FlowJo v.10 (Tree Star Inc., Ashland, OR, USA). Single-color stained samples, fluorescence-minus-one samples and isotype controls were used for compensation, gating and for background correction (Supplementary Figure S2). Gates to include singlets, living cells, lymphocytes, CD3^−^/CD3^+^ cells and further subpopulations of CD3^−^ and CD3^+^ T cells were defined (Supplementary Figure S2). Frequency of subsets expressing different markers was analyzed for total T cells (all CD3^+^ cells), CD4^+^ and CD8^+^ T cells (both gated from CD3^+^ cells).

### Cytokine analysis by multiplex assay

Fluorescent bead-based multiplex immunoassay was used to measure 26 cytokine/chemokine concentrations in BPH tissue supernatant collected during processing (*n* = 31). MILLIPLEX MAP Human Cytokine/Chemokine Premixed 26 Plex (Millipore Corporation, Temecula, CA, USA) was used according to manufacturer's protocol and as described before [32–34] and included: eotaxin/CCL11, G-CSF, GM-CSF, IFN-α2, IFN-γ, IP-10/CXCL10, MCP-1/CCL2, MIP 1-α/CCL3, MIP1-β/CCL4, TNF-α, TNF-β, IL-1α, IL-1β, IL-2, IL-3, IL-4, IL-5, IL-6, IL-7, IL-8/CXCL8, IL-10, IL-12p40, IL-12p70, IL-13, IL-15 and IL-17A. Analysis was done with Luminex IS 2.3 software (Luminex Corp., Austin, TX, USA) on a MAGPIX (Luminex xMAP Technology, Luminex Corp).

### Statistical analysis

Collected data was analyzed and displayed in Prism 6 (GraphPad, San Diego, CA, USA) and Microsoft Excel 14.5 (Microsoft Corp., Redmond, WA, USA). Non-parametric Mann-Whitney U-test was used to determine significant differences between PB and BPH tissue and also when comparing patient subgroups. Significance levels were set to *p≤0.05, **p≤0.01, ***p≤0.001 and ****p≤0.0001. Non-parametric Spearman's rank correlation coefficient was used to determine statistical correlation between different parameters. Nonlinear regression (least squares ordinary fit and robust fit straight line) was used to plot parameters.

## SUPPLEMENTARY FIGURES


