# Supplementary material for: Progression of benign prostatic hyperplasia is associated with pro-inflammatory mediators and chronic activation of prostate-infiltrating lymphocytes
Source: Oncotarget. 2016 Mar 14;7(17):23581–93. doi: 10.18632/oncotarget.8051 (PMC5029649; doi:10.18632/oncotarget.8051)
Supplement: Supplementary file 1 [file oncotarget-07-23581-s001.pdf]

SUPPLEMENTARY FIGURES

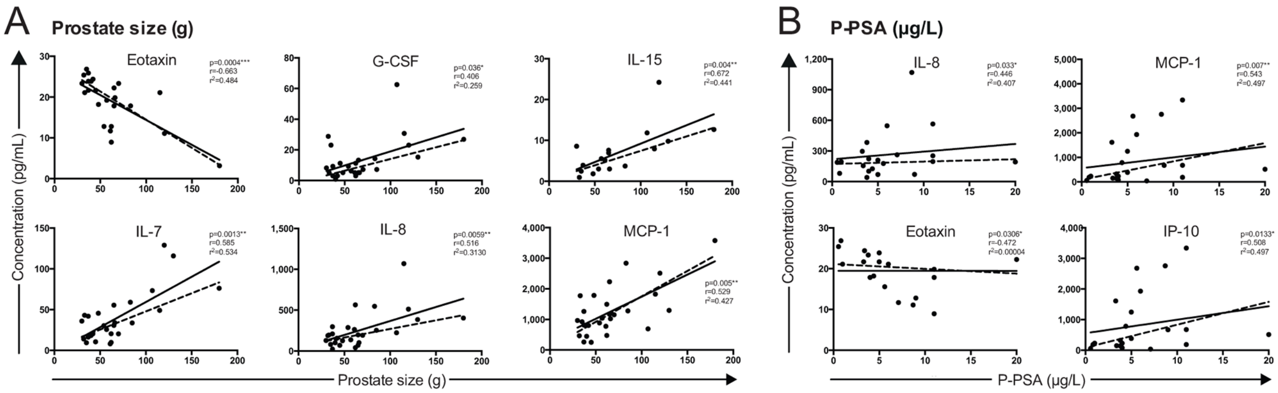

Supplementary Figure S1: Correlations between soluble proteins in supernatants of benign prostatic hyperplasia (BPH) tissue processing and clinical parameters. A. Prostate size of BPH patients correlates with several soluble proteins. **B.** Plasma prostate-specific antigen (p-PSA) levels in BPH patients correlates with several soluble proteins. The X-axis was cut at 20 μg/L resulting in two values not being shown but taken into account when calculating regression and correlation. Nonlinear regression is presented with r^2 (dashed line presents robust fit and straight line presents least squares (ordinary) fit), Spearman's correlation is presented by p-value and r. Significances are presented as *p≤0.05, **p≤0.01, and ***p≤0.001.

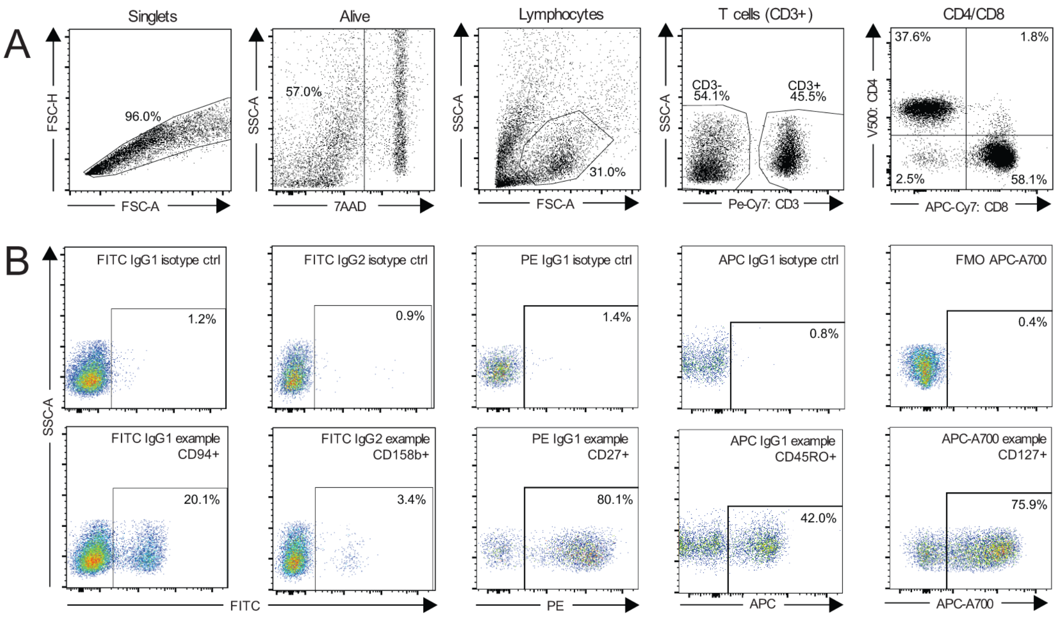

Supplementary Figure S2: Gating strategy and controls used for gating and correction of background. A. General gating strategy for analyzed characterization data. Singlets (forward scatter (FSC) height *vs*. area), viability (based on 7AAD), lymphocytes (FSC *vs*. side scatter (SSC)), CD3 expression and then further subpopulations such as CD4 and CD8 were plotted and analyzed. **B.** Representative plots of isotype controls and fluorescence-minus-one (FMO) with an example of a corresponding population (all gated from $CD3^+$).
